# Semi-metals as potential thermoelectric materials

**DOI:** 10.1038/s41598-018-28043-3

**Published:** 2018-06-29

**Authors:** Maxime Markov, Xixiao Hu, Han-Chun Liu, Naiming Liu, S. Joseph Poon, Keivan Esfarjani, Mona Zebarjadi

**Affiliations:** 1 Department of Electrical and Computer Engineering, University of Virginia, Charlottesville, Virginia 22904 USA; 2 Department of Physics, University of Virginia, Charlottesville, Virginia 22904 USA; 3 Department of Materials Science and Engineering, University of Virginia, Charlottesville, Virginia 22904 USA; 4 Department of Mechanical and Aerospace Engineering, University of Virginia, Charlottesville, Virginia 22904 USA

## Abstract

The best thermoelectric materials are believed to be heavily doped semiconductors. The presence of a band gap is assumed to be essential to achieve large thermoelectric power factor and figure of merit. In this work, we propose semi-metals with large asymmetry between conduction and valence bands as an alternative class of thermoelectric materials. To illustrate the idea, we study semi-metallic HgTe in details experimentally and theoretically. We employ *ab initio* calculations with hybrid exchange-correlation functional to accurately describe the electronic band structure in conjunction with the Boltzmann Transport theory to investigate the electronic transport properties. We calculate the lattice thermal conductivity using first principles calculations and evaluate the overall figure of merit. To validate our theoretical approach, we prepare semi-metallic HgTe samples and characterize their transport properties. Our first-principles calculations agree well with the experimental data. We show that intrinsic HgTe, a semimetal with large disparity in its electron and hole masses, has a high thermoelectric power factor that is comparable to the best known thermoelectric materials. Finally, we propose other possible materials with similar band structures as potential candidates for thermoelectric applications.

## Introduction

Since its discovery in 1821, thermoelectricity remains in the center of interests of the scientific community. Thermoelectric effect (Seebeck effect) refers to direct conversion of thermal to electrical energy in solids and can be used for power generation and waste heat recovery^1–4^. Despite their clean, environmentally friendly and reliable performances, thermoelectric modules are only used in niche applications such as in powering space probes. The main obstacle preventing thermoelectric technology to be widely used on a mass market today is its relatively low efficiency^5^.

The thermoelectric efficiency is an increasing function of the material’s dimensionless figure of merit $zT=\frac{{S}^{2}\sigma }{\kappa }T$ where *S* is the Seebeck coefficient, *σ* is the electrical conductivity, *κ* is the thermal conductivity, and *T* is the absolute temperature. The first two quantities can be combined together into the thermoelectric power factor *P*_*F*_ = *S*^2^*σ* describing electronic transport, in contrast to the thermal conductivity, *κ*, related to thermal transport. The power factor is often used as a guide to preselect the class of potential thermoelectric materials. Indeed, metals have highest electrical conductivity but suffer from a low Seebeck coefficient. The reason for their low Seebeck coefficient is the symmetry of the density of states around the chemical potential. The number of hot electrons above the chemical potential in a metal is roughly the same as the number of cold empty states below the chemical potential. As a result under a temperature gradient, the number of electrons diffusing from the hot side to the cold side, is approximately equal to the number of cold electrons diffusing from the cold side to the hot side. The same problem does not exist in semiconductors due to the presence of a band gap allowing only one type of the carriers to diffuse. Typical Seebeck coefficient of semiconductors is two orders of magnitude larger than metals. Ioffe first noticed this advantage of semiconductors^6^ and paved the way for many successful demonstration of doped semiconductors with high zT values. Later, several research groups including Chasmar & Stratton^7^ and Sofo & Mahan^8^ studied the effect of band gap on thermoelectric properties of materials employing two-band toy models for electronic structure and reached the conclusion that best thermoelectric materials must have band gap greater than 6–10 *k*_*B*_*T*. Today heavily doped semiconductors are the main focus of the thermoelectric society^8,9^. While opening a band gap is a proven way of increasing the Seebeck coefficient, in this article we show that to have a large Seebeck coefficient, a band gap is not a must. What needed is an asymmetric density of states which could be achieved also in semi-metals with slight overlap of electrons and holes bands but with large asymmetry in the electron and hole effective masses.

We turn our attention to semi-metallic HgTe whose properties are in the transition region between semiconductors and metals. HgTe has a very high electron/hole effective mass ratio ${m}_{e}/{m}_{h}\simeq 0.1$^10^ which results in large values of the Seebeck coefficient between −90 *μV*/*K*^11^ and −135 *μV*/*K*^12^ at room temperatures which is similar to the Seebeck coefficient of heavily doped semiconductors with a band gap. The carrier concentration of intrinsic HgTe is only 10^16^–10^17^ *cm*^−3^ which is much smaller than a metal or a typical good heavily-doped semiconductor thermoelectric. However, the large electron mobility in HgTe (*μ* > 10^4^ *cm*^2^/*V*.*s*)^10^ makes up for its low carrier concentration and as a result, the electrical conductivity of an intrinsic sample is relatively large and is about *σ* = 1700 *S*/*cm*^11,12^ at room temperatures. The large electron mobility is partly due to the small effective mass of the electrons and partly because of the absence of dopants. The mobility of a heavily doped semiconductor is limited by ionized impurity scattering which is not the case in an intrinsic semi-metal. The experiment reveals that intrinsic HgTe is a high power factor material with *P*_*F*_ = 14–31 *μ*W cm^−1^ K^−2^ at T = 300 K^11,12^ that is comparable to well-known thermoelectric materials such as SnSe (${P}_{F}\simeq 10$ *μ*W cm^−1^ K^−2^), PbTe_1−*x*_Se_*x*_ (${P}_{F}\simeq 25$ *μ*W cm^−1^ K^−2^) and Bi_2_Te_3_ (${P}_{F}\simeq 50$ *μ*W cm^−1^ K^−2^) at their zT maximum^13^. Apart from having a good electrical transport properties, mercury telluride is a good thermal insulator with *κ* = 2.1 W/mK^11,14^ at T = 300 K. The overall zT of intrinsic single crystal without any optimization is between 0.4 to 0.5 and is comparable with most good thermoelectric materials at room temperature.

The most recent theoretical study of HgTe concludes that semi-metallic HgTe (zinc-blende phase) is a poor thermoelectric material with room temperature zT values close to zero in intrinsic samples^15^ and emphasize the superior thermoelectric performance of a high pressure semiconducting cinnabar phase^15,16^. However, these studies rely on a standard GGA-PBE exchange-correlation functional to describe the electronic structure of a semi-metallic HgTe which fails to reproduce the asymmetry in the density of states near the Fermi level. Moreover, the use of the same constant relaxation time at different doping concentrations results in an erroneous conclusion that the electrical conductivity always grows with the increase of doping. On the contrary, the experimental data shows the drastic decrease of the electrical conductivity with doping in *p*-type samples of HgTe^11^.

In this work, we perform a combined theoretical and experimental study of thermoelectric properties of HgTe at high temperatures. To address the above mentioned issues, we employ *ab initio* calculations with hybrid exchange-correlation functional in conjunction with the Boltzmann Transport theory with energy dependent relaxation times obtained from the fitting of experimental electrical conductivity. We do not attempt to optimize the thermoelectric properties of HgTe using nanostructuring, alloying or slight doping. Instead, we attempt to develop a platform based on first principles calculations to study its transport properties and to make a case for semi-metals as potential candidates for thermoelectric applications.

## Results and Discussion

### Electrical transport

The electronic band structure of zinc-blende HgTe has been extensively studied over the past decade^17–20^. It has been shown that *ab initio* calculations with standard LDA and GGA exchange-correlation functionals can not accurately describe the band structure of HgTe. To achieve a good agreement with experiment, one must perform either GW calculations^18,19^ or use a hybrid functional^17,20^ where a portion of exact Fock exchange interaction is introduced into a standard exchange-correlation functional.

**Figure 1:**
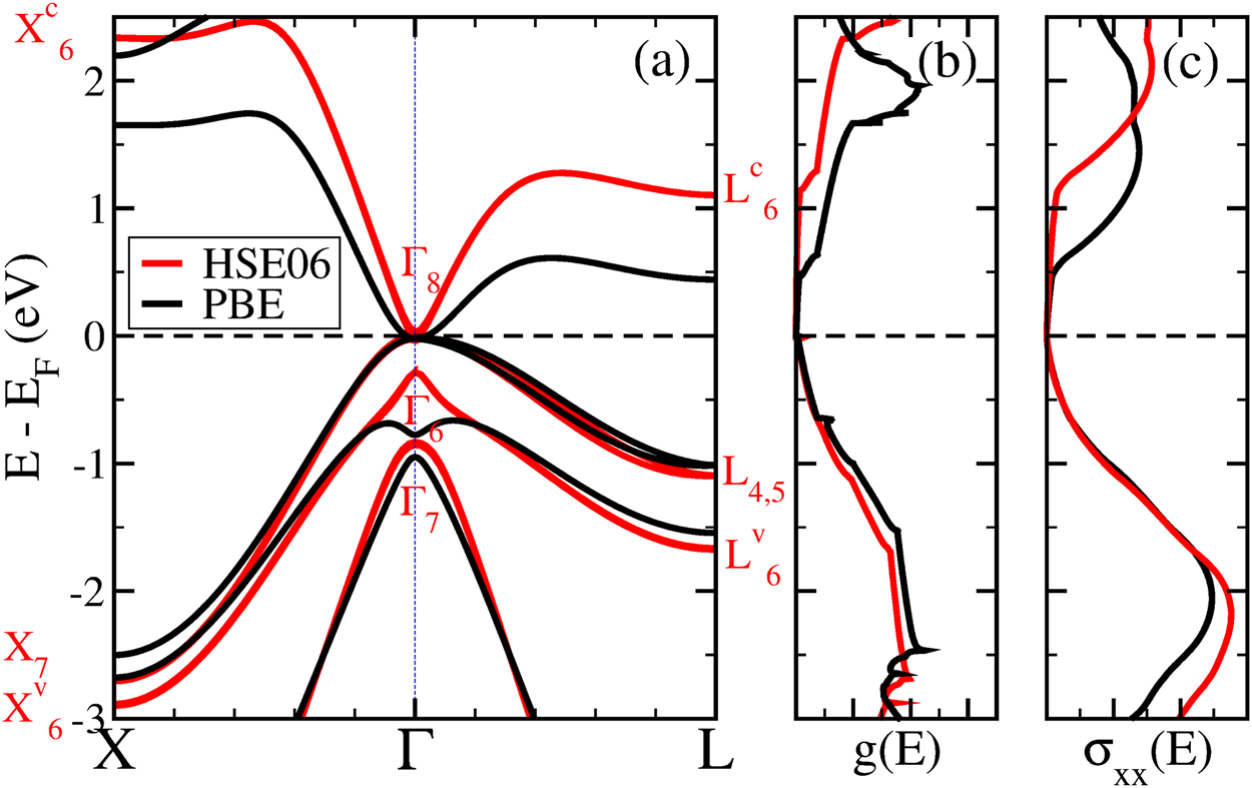
Electronic band structure (panel a), density of states *g*(*E*) (panel b) and differential conductivity *σ*_*xx*_(*E*) (panel c) calculated using PBE (black curves) and HSE06 (red curves) exchange-correlation functionals. Energy levels from the latter calculation are labeled according to their symmetries.

In Fig. 1(a), we compare the electronic band structures calculated using GGA-PBE^21^ (black curves) and hybrid-HSE06^22^ (red curves) exchange-correlation functionals and summarize the theoretical and experimental band edges, E, and spin-orbit splittings, Δ, at Γ, L and X high symmetry points in Table 1. First, we note that the HSE06 calculation predicts the correct level ordering Γ_7_, Γ_6_, Γ_8_^18,20^ that is consistent with experiment^23^ in contrast to the GGA-PBE calculation where the Γ_6_ and Γ_7_ bands are reversed. Second, the band energies obtained with the hybrid functional are in excellent agreement with experiment. For instance, the inverted band gap ${E}_{g}={E}_{{{\rm{\Gamma }}}_{6}}-{E}_{{{\rm{\Gamma }}}_{8}}=-\,0.27$ eV and spin-orbit splitting ${{\rm{\Delta }}}_{0}={E}_{{{\rm{\Gamma }}}_{8}}-{E}_{{{\rm{\Gamma }}}_{7}}=0.89$ eV at Γ differ from their experimental values only by 0.02 eV. Third, the effective mass of the lowest conduction band is significantly reduced from *m*_*e*_ = 0.18 *m*_0_ in GGA-PBE to *m*_*e*_ = 0.04 *m*_0_ in HSE06 in the [100] direction, whereas the effective mass of the top valence bands remains essentially unchanged *m*_*h*_ = 0.29 *m*_0_ in GGA-PBE to *m*_*h*_ = 0.33 *m*_0_ in HSE06. Thus, HgTe is a material with a very high electron-hole effective mass ratio.

**Table 1:** Energy band edges, *E*, and spin-orbit splittings, Δ, at Γ, *L* and X high symmetry points calculated with the GGA-PBE and hybrid-HSE06 functionals.

	GGA-PBE	HSE06	Expt.
*E*_Γ_ = E(Γ_6_) − E(Γ_8_)	−0.93	−0.27	−0.29^23^, −0.30^53^
Δ_Γ_ = E(Γ_8_) − E(Γ_7_)	0.76	0.89	0.91^23^
*E*_*L*_ = E(${L}_{6}^{c}$) − E(*L*_4,5_)	1.45	2.19	2.25^53^
Δ_*L*_ = E(*L*_4,5_) − E(${L}_{6}^{v}$)	0.54	0.56	0.62^53^, 0.75^54^
*E*_*X*_ = E(${X}_{6}^{c}$) − E(*X*_7_)	4.15	5.02	5.00^54^
Δ_*X*_ = E(*X*_7_) − E(${X}_{6}^{v}$)	0.19	0.22	0.1–0.2^54^

Experimental results from the literature are shown.

Finally, the electronic properties of HgTe near the Fermi level are defined by the region of the Brillouin zone close to the Γ point, where the bands have a low degeneracy. This low degeneracy in combination with a small electron effective mass in HSE06 calculation results in a small density of states of conduction bands. The asymmetry between the conduction and valence bands is clearly seen in both, the density of states *g*(*E*) and the differential conductivity *σ*_*xx*_(*E*), as can be seen in Fig. 1.

**Figure 2:**
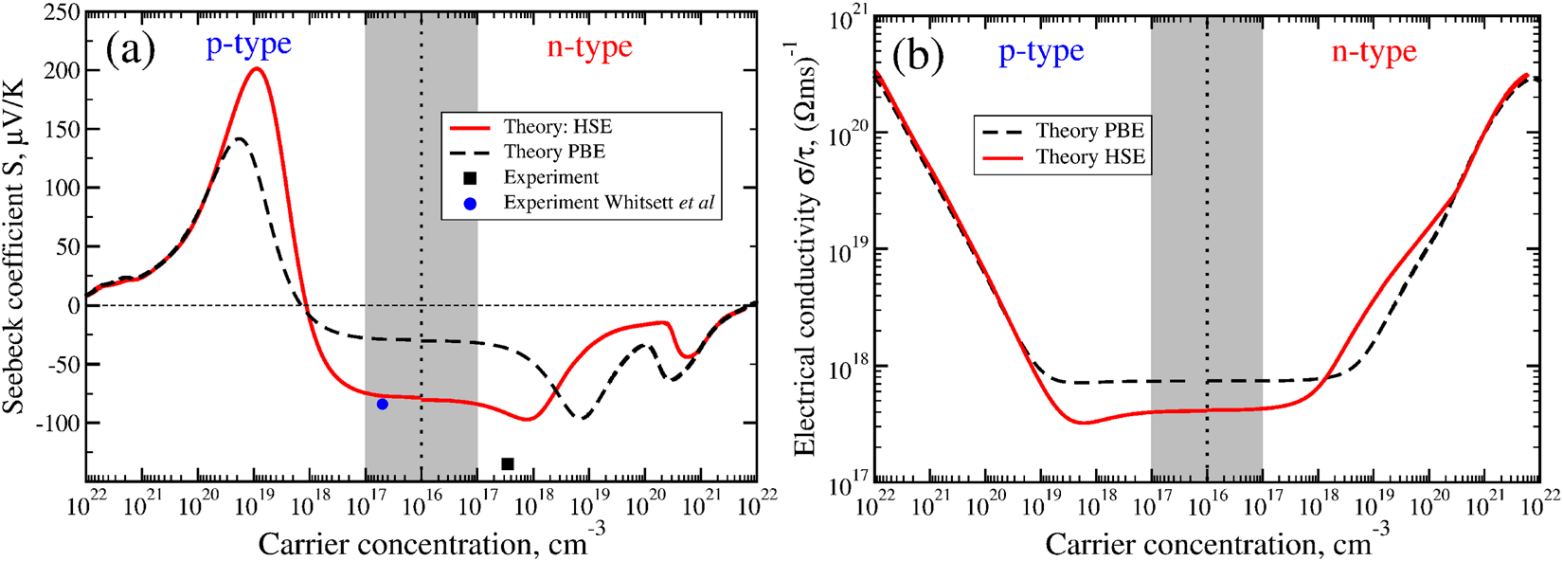
The Seebeck coefficient (panel (a)) and the electrical conductivity (panel (b)) as a function of carrier concentration for *p*-type and *n*-type samples at T = 290 K calculated with GGA-PBE (black dashed line) and hybrid-HSE06 (solid red line) functionals. Experimental data from Whitsett *et al*.^11^ is shown by a blue circle and experimental data measured in this work is shown by black square. The x-axis is the doping density which is the difference between the number of electrons at a given Fermi-level and the number of valence electrons. The doping density of the experimental data is estimated from the Hall coefficient (*n* = 1/*R*_*H*_), assuming a Hall factor of 1. The shaded region corresponds to the intrinsic concentration range.

In Fig. 2(a), we show the Seebeck coefficient as a function of doping concentration for *p*- and *n*-types of doping at T = 290 K calculated using the constant relaxation time approximation. Our results with the GGA-PBE functional agree well with the previous calculation of Chen *et al*.^15^ done with the same exchange-correlation potential. As it is expected from the band structure calculations, one can see a noticeable change in the magnitude of the Seebeck coefficient due to the increase of the electron-hole effective mass ratio in HSE06 calculation. For instance, the maximum of the Seebeck coefficient is increased from 142 *μ*V/K to 202 *μ*V/K and is slightly shifted towards the lower doping concentrations from 2·10^19^ cm^−3^ to 9·10^18^ cm^−3^. In intrinsic and low doped samples (up to 10^17^ cm^−3^), the Seebeck coefficient remains constant but also has a sufficiently higher magnitude of −81 *μ*V/K with HSE06 instead of −31 *μ*V/K with GGA-PBE. Our HSE06 result is in good agreement with experimental result −91 *μ*V/K (blue circle) reported by Whitsett *et al*.^11^ for p-type sample. However, our measurements in n-type HgTe sample with *n* = 3.5·10^17^ cm^−3^ doping concentration show much larger values of the Seebeck coefficient of −136 *μ*V/K (black square).

The constant relaxation time theory, does not allow to compute the electrical conductivity but only its ratio to the unknown relaxation time $\frac{\sigma }{\tau }$. As can be seen in Fig. 2(b), this ratio varies slowly at low doping concentrations and grows rapidly at high doping concentrations. However, one would expect a different behavior for the electrical conductivity at least in the high doping concentration region where a strong charged carrier scattering limits the mobilities. The electrical conductivity of HgTe is highly sensitive to the number of impurities and defects in the sample, an experimentally unknown parameter. Therefore, one has to use the number of defects as a fitting parameter to estimate the electrical conductivity. To further investigate the behavior of the electrical conductivity and the Seebeck coefficient, we introduce phenomenological scattering rates and fit their coefficients to reproduce our experimental electrical conductivity data in *n*-type sample.

**Figure 3:**
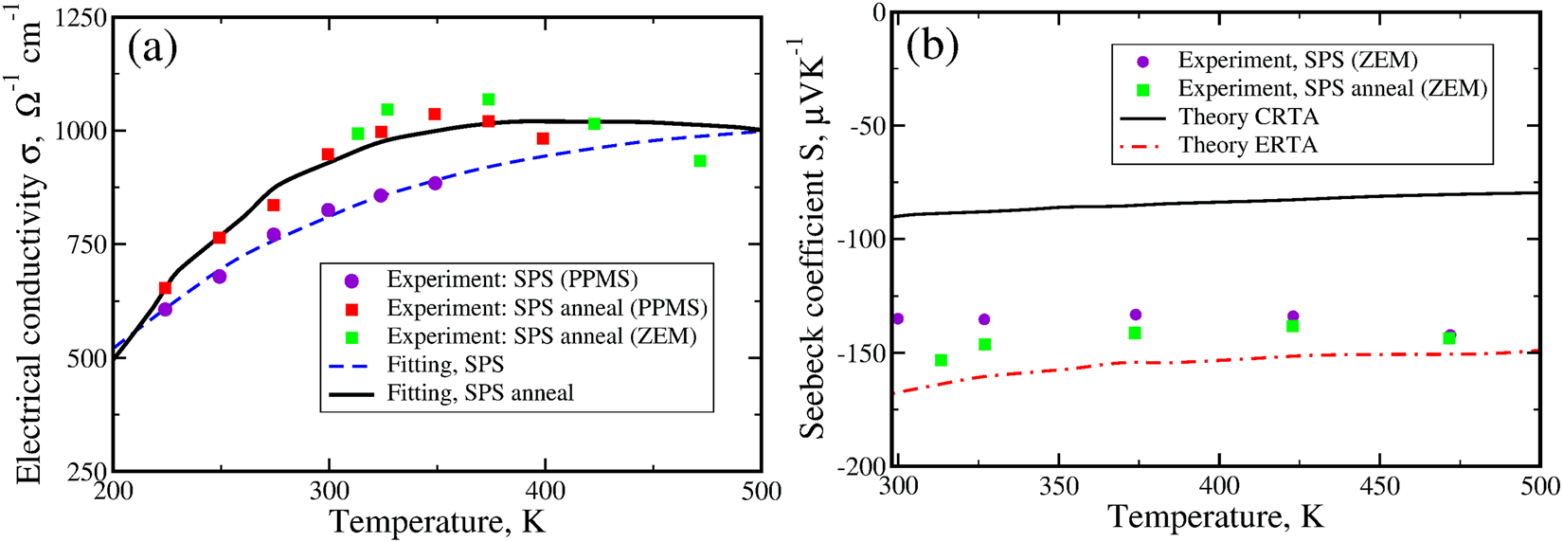
Panel (a): Temperature variation of the electrical conductivity *σ* measured in the experiment in *n*-type samples before (violet circles) and after (red and green squares) annealing. The fitting curves are shown by dashed blue and solid black lines respectively. Panel (b): Temperature variation of the Seebeck coefficient *S* for *n*-type samples measured in experiment (violet circles and green squares). The theoretical Seebeck coefficients calculated in the CRTA and in the ERTA are shown by black solid and red dashed dotted lines respectively. We have used ZEM and PPMS systems for the measurements.

Figure 3(a) show our experimental data obtained using the four-terminal probe method^24^ in the samples prepared using the spark plasma sintering (SPS) technique. Two sets of measurements before (violet circles) and after (red and green squares) annealing have been performed. As expected, annealing improves the electrical conductivity^11,25^ which reaches its maximum value of *σ* = 1036 (Ω cm)^−1^ at T = 350 K and then starts monotonically decreasing at higher temperatures. We notice that our results are much lower than the electrical conductivity *σ* = 1700 Ω^−1^ cm^−1^ measured in the intrinsic samples at T = 300 K^11,12^. These intrinsic samples were prepared by multiple annealing of the originally *p*-type samples in the presence of Hg gas^11,12^. However, in this work we do not follow this procedure due to the extreme toxicity of mercury.

We fit the measured electrical conductivity using *ab initio* data for the differential conductivity *σ*_*xx*_(*E*) and the density of states *g*(*E*) obtained with the hybrid-HSE06 functional and phenomenological energy dependent scattering rates accounting for the acoustic deformation potential, polar optical and ionized impurity scattering rates^26^. Details of the considered scattering rates are described in Supplementary information. We then recalculate the Seebeck coefficient using the obtained scattering rates and find that its magnitude is increased about 2 times with respect to the constant relaxation time approximation (CRTA). The energy dependent relaxation time approximation (ERTA) results in Seebeck coefficient values that are closer to the experimentally measured ones. Therefore we conclude that the difference between the CRTA calculations (Fig. 2a) and experimental values is a result of the energy dependence of the scattering rates. Although the Seebeck coefficient is not as sensitive as the conductivity to the relaxation times, this example demonstrates that CRTA results could be misleading even in calculation of the Seebeck coefficient. The importance of ERTA has been discussed before. For example, see refs^27–29^.

The temperature variation of the Seebeck coefficient calculated in the CRTA (black solid lines), the ERTA (red dashed dotted line) and measured in experiment are shown in Fig. 3(b). As one can see, both the theoretical and experimental Seebeck coefficients remain almost temperature independent in the studied temperature range between 300 and 500 K. The melting point of HgTe is 943 K. Thus, in principles, investigation of the thermoelectric properties could be extended to much higher temperatures than reported in this work. We found that at temperatures above 500 K, Hg tends to segregate and evaporate in a ZEM-3 environment that we used for transport measurements. Thus we limit our measurements to up to 500 K.

Our study reveals that for the accurate description of the electrical transport properties of HgTe, one needs to accurately reproduce the electron-hole effective mass ratio that can not be achieved using standard LDA or GGA exchange-correlation functionals. Moreover, we find that the inclusion of energy dependent scattering rates changes the magnitude of the Seebeck coefficient drastically. The latter has been unexpected since, according to the common believe^30^, the CRTA reproduces well the behavior of the diffusion part of the Seebeck coefficient. The magnitude of the Seebeck coefficient of HgTe is an order of magnitude higher than the one in typical metals and close to the typical values of narrow-gap semiconductors. That is explained by the the low effective mass and low degeneracy of the conduction band near the Fermi level. We then conclude that the presence of a band gap is not essential for obtaining large Seebeck coefficient values.

### Thermal transport

**Figure 4:**
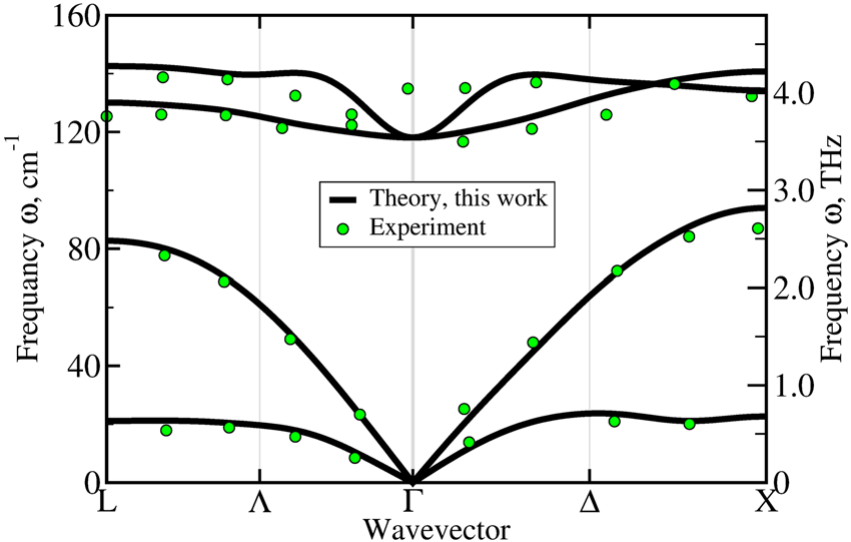
Theoretical phonon dispersion calculated using DFPT in this work (black curves) compared to the inelastic neutron scattering data (green circles)^33,34^.

Now, we turn our attention to the thermal transport properties of HgTe. First, we investigate the lattice dynamics by calculating the phonon spectrum along the high symmetry directions. The phonon dispersion is shown in Fig. 4 and is in an excellent agreement with previous theoretical results^16,31,32^ as well as with available data from the inelastic neutron scattering experiments^33,34^ (green circles). In our calculations we do not take into account the non-analytical correction to split the optical phonons at Γ point. However, this correction should not strongly affect the thermal conductivity since the contribution is usually small due to the low group velocities of optical phonons. Our theoretical frequencies for optical phonons *ω*_*O*_(Γ) = 118 cm^−1^ agree well with the Raman spectroscopy data for the transverse optical phonons *ω*_*TO*_(Γ) = 116 cm^−1 ^^35^.

To further validate the vibration spectrum, we calculated the elastic constants *C*_*ij*_. As shown in Table 2, the difference between our theoretical results and experiment does not exceed 4%. Then, we compare the sound velocities in [100] direction obtained from the elastic constants, from the slopes of acoustic branches near the Γ point and experimental data in Table 3. The largest differences with the experiment, 7.1% and 2.5% for the transverse (TA) and longitudinal (LA) sound velocities respectively, are found for the evaluation of sound velocities from the slopes of acoustic phonons.

**Table 2:** Elastic constants *C*_*ij*_ (GPa) calculated in the present work and compared with other theoretical calculations^55,56^ and experiment^57^.

	*C*_11_, GPa	*C*_12_, GPa	*C*_44_, GPa
Present	57.3	41.0	22.0
Experiment	59.7^57^	41.5^57^	22.6^57^
2*Other	56.3^55^	37.9^55^	21.2^55^
	67.4^56^	45.7^56^	30.0^56^

**Table 3:** The longitudinal *v*_*L*_ and transverse *v*_*T*_ sound velocities (m/s) in [100] direction calculated in the present work from the elastic constants, slopes of acoustic phonons and experiment.

	*vL*, m/s	*vT*, m/s
Elastic constant	2655	1645
Slope	2747	1504
Experiment	2680	1620

**Figure 5:**
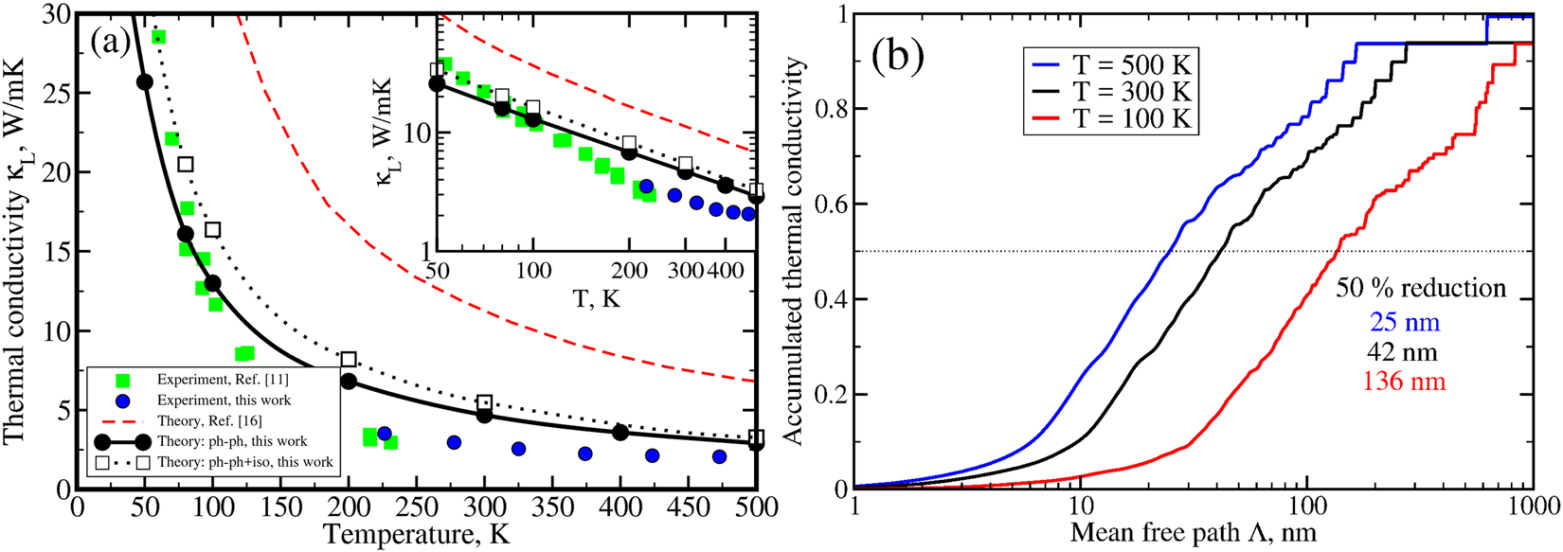
Panel (a): Temperature dependence of the thermal conductivity calculated with account for anharmonic three-phonon processes only (black dashed line) and with addition of isotopic disorder scattering (black solid line); green squares - experimental data from Whitsett *et al*.^11^; blue circles - our experimental data; dashed red curve - previous computational result from refs^16,32^. The thermal conductivity data in log-log scale are shown in the insert. Panel (b): Accumulated thermal conductivity as a function of phonon mean free path Λ at T = 100 K (blue curve), 300 K (black curve) and 500 K (red curve). Horizontal dotted line denotes 50% thermal conductivity reduction.

Figure 5a summarizes the theoretical and experimental thermal conductivity obtained in this work as well as those reported by other groups. We perform the lattice thermal conductivity calculations by *exactly* solving the Boltzmann Transport Equation (BTE). First, we include only the intrinsic three-phonon anharmonic scattering (dotted black curve). We obtain the lattice thermal conductivity that is much lower than the previous *ab initio* calculations (red dashed curve)^16^. For instance, we get *κ*_*L*_ = 5.48 W/mK instead of *κ*_*L*_ = 10.46 W/mK in Ref.^16^. Our theoretical values are still higher than ones measured in experiment *κ*_*L*_ = 2.9 W/mK (this work) or *κ*_*L*_ = 2.14 W/mK (ref.^11^). This discrepancy can not be attributed to the extrinsic sources of scattering such as the impurity scattering since the experimental data for the *p*-type samples with doping concentration between 10^16^–10^18^ cm^−3^ show essentially the same thermal conductivity^11^. The addition of isotopic disorder scattering significantly decreases the thermal conductivity mainly at low temperatures (black solid curve) whereas at high temperatures the isotopic scattering plays a minor role. At room temperature we get *κ*_*L*_ = 4.68 W/mK that is still higher than experimental values.

While we capture the low temperature trend, we attribute the disagreement between experiment and theory at higher temperatures to some intrinsic scattering mechanism which has not been taken into account in our calculations. Since only 3-phonon processes are included in the calculation of the thermal conductivity, the decay stays proportional to 1/T at high T. As can be seen in the insert of Fig. 5a, our experimental data show a different trend at *T* > 300 K. The slope changes and the thermal conductivity becomes almost flat and temperature independent. We assume that four-phonon anharmonic processes or higher order three phonons are important because of the deviations of *κ*(*T*) from the 1/T behavior. Thus, the lattice thermal conductivity of HgTe should be subject to further investigation.

In Fig. 5b, we analyze the accumulated lattice thermal conductivity *κ*_*L*_(Λ) as a function of phonon mean free path Λ (see supplementary material for details) at three different temperatures T = 100 K (blue curve), 300 K (black curve), 500 K (red curve). As one can see, the thermal conductivity is mainly cumulated below 1 micron and the mean free paths become shorter when temperature is increased. The accumulated function can be used to predict the effective size *L* of a nanostrucure necessary to reduce the thermal conductivity and, thus, increase the thermoelectric performance of a material. Indeed, phonons with mean free paths larger than *L* are scattered by sample boundaries and their contribution to the thermal conductivity is suppressed. The horizontal dotted line denotes a 50% reduction of thermal conductivity. It is found to be *L* = 136 nm at T = 100 K, *L* = 42 nm at T = 300 K and *L* = 25 nm at T = 500 K.

### Thermoelectric performance

**Figure 6:**
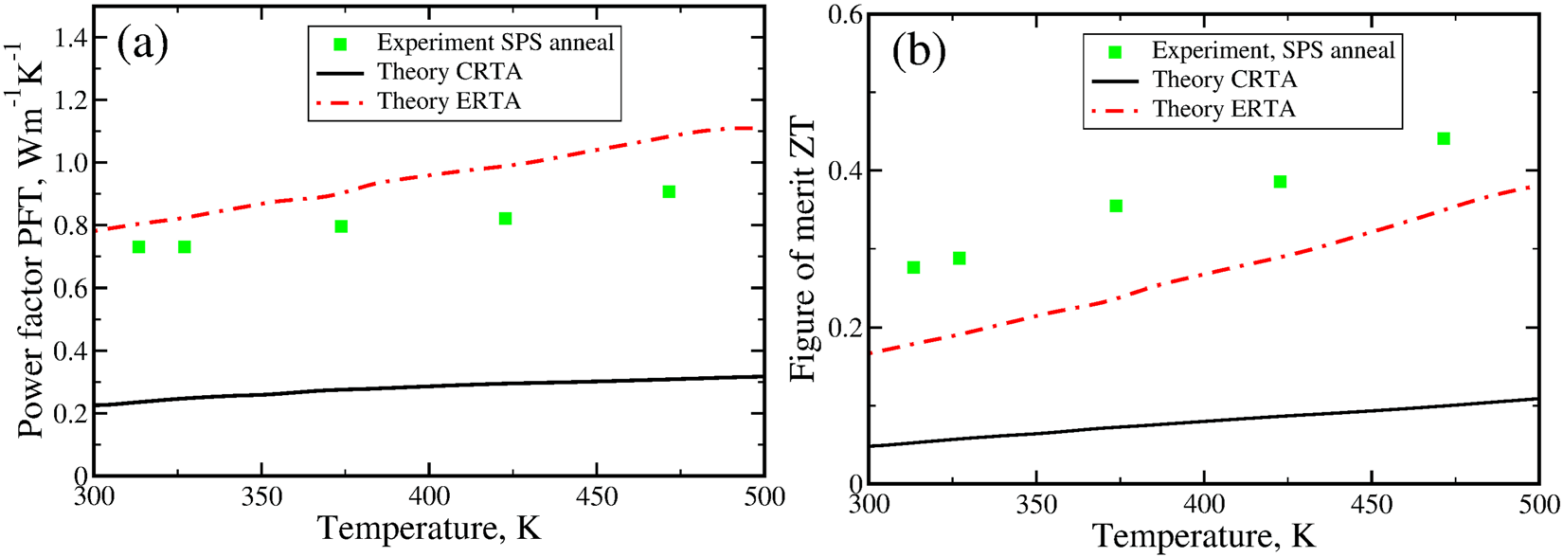
Temperature variation of the power factor *PFT* = *S*^2^*σT* (panel a) and thermoelectric figure of merit *zT* (panel b) measured in the experiment (green squares) and calculated in the CRTA (black solid line) and in the ERTA (red dashed dotted line).

Finally, we evaluate the overall thermoelectric power factor *PFT* = *S*^2^*σT* based on our experimental and theoretical data in Fig. 6(a). As one can see, HgTe possess a high power factor which grows with temperature linearly from 0.8 W m^−1^ K^−1^ at T = 310 K to 0.9 W m^−1^ K^−1^ at T = 475 K. Our theoretical values obtained in the ERTA slightly overestimate the experimental power factor but show the same temperature dependence reaching 1.0 W m^−1^ K^−1^ at T = 500 K. The CRTA underestimates the magnitude of the Seebeck coefficient and results in a low power factor around 0.2 W m^−1^ K^−1^.The figure of merit also increases linearly since thermal conductivity is relatively unchanged in this temperature range.

While zT values reported here are small. We would like to emphasize that this is not an optimized sample. One can increase the zT values by many different techniques. For example, further increase in the electrical conductivity (a factor of two) is expected after annealing in Hg gas with relatively unchanged Seebeck coefficient and thermal conductivity values^11,12^. As mentioned earlier we avoid this process due to both toxicity of Hg gas and the fact that optimization of the thermoelectric properties of HgTe is not the subject of this work. One can also implement nanostructuring to further reduce the thermal conductivity, a technique that is routinely performed to optimize the thermoelectric figure of merit. Similarly, slight doping (tunning of the chemical potential) and slight alloying could be used to further optimize the performance of semi-metallic HgTe. For example, alloying with cadmium could lower the thermal conductivity and still preserves the semi-metallic nature of the HgTe for small molar fractions of cadmium (*x* < 0.1).

Finally, we would like to point to an interesting observation. In most heavily-doped semiconductors, the thermoelectric transport properties are inter-related. An increase in the electrical conductivity usually results in an increase in the thermal conductivity (via its electronic part) and results in a decrease in the Seebeck coefficient. Both of which are unwanted for obtaining high thermoelectric figure of merit. In contrast, within the semi-metallic HgTe samples we tested: the originally purchased ingots, the mechanically-milled and SPS samples, and the annealed samples, all showed similar Seebeck coefficient and thermal conductivity values but very different electrical conductivity values. The electrical conductivity value of the annealed sample was increased by almost a factor of three compared to the original ingot (See supplementary information). This feature could be a very useful one enabling tuning the thermoelectric figure of merit.

### Other prospective semi-metallic TE materials

While this work is focused on HgTe, we would like to emphasize that HgTe was taken only as an illustration. The more important point here is to introduce an alternative class of thermoelectric materials. Our work demonstrates that large thermoelectric power factors can be achieved even in the absence of an energy band gap. This is not unique to the case of HgTe. Other semi-metals with large asymmetry between electrons and holes masses also hold promise for thermoelectric applications. Many of these materials are topologically non trivial and have been studied in other fields. For example, TaAs_2_ has been studied recently by Luo *et al*.^36^. They found that the three weak topological indices of TaAs_2_ are non-trivial. The material demonstrates large density of states below the Fermi level and small ones above the Fermi level. Another example is Y_2_Ir_2_O_7_^37^ which is shown to be a topological semi-metal with heavy electrons. Cd_3_As_2_ is another material with broken-symmetry three-dimensional topological Dirac semi-metal system with strong spin-orbit coupling that has a large asymmetry between its electrons and holes^38^. Ge_1−*x*_Sn_*x*_ alloys are shown to have several electronic phases. One of the phases is shown to be a topological semi-metal with a band structure similar to that of HgTe^39^. Other examples include but are not limited to group V semi-metals^40^, HgCr_2_Se_4_^41^, and Pt_3_Sn^42^. These semi-metallic materials with topological properties which is often due to a strong spin-orbit coupling also possess a small thermal conductivity due to the heavy mass of some of their constituents. These compounds therefore warrant systematic study as a new class of high-zT materials using state-of-the-art computational methods.

## Conclusion

We proposed semi-metals with large asymmetry between electrons and holes as potential thermoelectric candidates. As a test candidate, we have investigated, both experimentally and theoretically, the electrical and thermal transport properties of HgTe at high-temperatures between 300 and 500 K. In contrast to a recent theoretical prediction, we have found that intrinsic HgTe is a good thermoelectric material in a low pressure semi-metallic zinc-blende phase as it has a high Seebeck coefficient and a low thermal conductivity. To explain the experimental data for the Seebeck coefficient, we accurately reproduced the electron-hole effective mass ratio by performing *ab initio* calculations with the hybrid-HSE06 exchange-correlation functional and taking into account the phenomenological scattering rates extracted from a fit to electrical conductivity. Finally, we performed the lattice thermal conductivity calculations by *exactly* solving the Boltzmann Transport Equation (BTE). We included three-phonon anharmonic scattering and isotopic disorder scattering processes. We attribute the disagreement between experiment and theory to some intrinsic scattering mechanism which has not been taken into account in our calculations.

## Methods

### Theoretical methods

Our theoretical calculations are based on density functional theory (DFT). For the electrical transport calculations, we use Vienna Ab-initio Simulation Package (VASP)^43,44^ combined with Boltzmann Transport Theory as implemented in Boltztrap code^30^. We use pseudopotentials based on the projector augmented wave method^45^ from VASP library with the generalized gradient approximation by Perdew, Burke and Ernzehof (GGA-PBE)^21^ and with a hybrid Heyd-Scuseria-Ernzehof (HSE06)^22^ exchange-correlation functionals. A plane wave kinetic cut-off of *E*_*cut*_ = 350 eV and Γ-centered k-point mesh of 8 × 8 × 8 were found to be enough to converge the total energy up to 5 meV^20,46^. These parameters were used for band structure calculations. We use a tetrahedron method for the Brillouin zone integration and the experimental lattice parameter of a = 6.460 Å. In our calculations, we take into account the spin-orbit coupling which is important to accurately reproduce the electronic band structure of HgTe. Transport calculations were performed on much denser DFT grids: 40 × 40 × 40 for PBE-GGA and 20 × 20 × 20 for HSE06. The details about the convergence with respect to DFT grid can be found in the Supplementary Material. To ensure the convergence of transport integrals in Boltztrap, we ues 20 times denser interpolated grid than we use in our ab initio calculations.

For the thermal transport calculations, we use Quantum Espresso^47^ package combined with D3Q code to calculate third-order anharmonic force constants using “2n + 1” theorem^48^ and to solve the Boltzmann transport equation for phonons variationally^49^. We use the norm-conserving pseudopotentials with the exchange-correlation part treated in the local density approximation by Perdew and Zunger (LDA-PZ)^50^. We use a cut-off energy of *E*^*cut*^ = 1360 eV (100 Ry), 8 × 8 × 8 k-points mesh to sample the Brillouin zone with Methfessel-Paxton smearing of *σ* = 0.068 eV (0.005 Ry). The equilibrium lattice parameter is found to be 6.431 *Å*. Spin-orbit coupling is not included in the calculations since it has a weak effect on vibrational properties of HgTe as has been pointed out by M. Cardona *et al*.^31^. Phonon frequencies and group velocities are calculated using the density functional perturbation theory (DFPT)^51^ on a 8 × 8 × 8 **q**-point grid centered at Γ. The third-order anharmonic constants are calculated on a 4 × 4 × 4 **q**-point grid in the Brillouin zone that amounts to 42 irreducible triplets. Both phonon harmonic and anharmonic constants are then interpolated on a dense 24 × 24 × 24 **q**-point grid necessary to converge the thermal conductivity calculations.

The detailed information about the charged carrier scattering rates obtained from the electrical conductivity fit and about the isotopic disorder scattering rates used in the lattice thermal conductivity calculation is reported in the supplementary material.

### Experimental methods

A 99.99% purity of HgTe ingot was purchased from 1717 CheMall Corporation for HgTe sample preparation and the density of the ingot was 7.82 ± 0.04 g/cm^3^ obtained by Archimedes’ principle. We crashed the ingot and milled it with a mortar and pestle for about 10 minutes to obtain fine powders. Later, they were consolidated into a 0.5″-diameter compact disk by using spark plasma sintering (SPS) method at 783 K, 50 MPa for 15 minutes. After SPS process, the density of the HgTe disk is increased to 7.98 ± 0.17 g/cm^3^, which is quite close to the theoretical fully-dense value of the HgTe density 8.13 g/cm^3^. For annealing preparation, the compact HgTe sample was sealed in an evacuated capsule, and it was situated in the middle of a furnace at 523 K for 5 days.

For the ingot samples, we used the machine to cut it into a rectangular-bar-shaped sample with the dimension of 2 × 4 × 8 mm^3^. For the SPS samples, due to their fragality, we hand-polished the disk into a rectangular-shaped bar of the same size as the ingot one instead of cutting them in the machine. The four-probe electrical conductivity and Seebeck coefficient measurements were performed in the helium atmosphere with a ZEM-3 equipment from Ulvac Tech., Inc. The Hall coefficient measurements were conducted in Quantum Design Versa-Lab. The thermal diffusivity experiments were carried out with a LFA 467 HyperFlash equipment from NETZSCH. The measured thermal diffusivity was then multiplied by the theoretical heat capacity^52^ *C*_*p*_(*T*) = *C*_*V*_(*T*) + 1.01·10^−2^ *T* where *C*_*V*_(*T*) was obtained from the Debye model.

**Figure 7:**
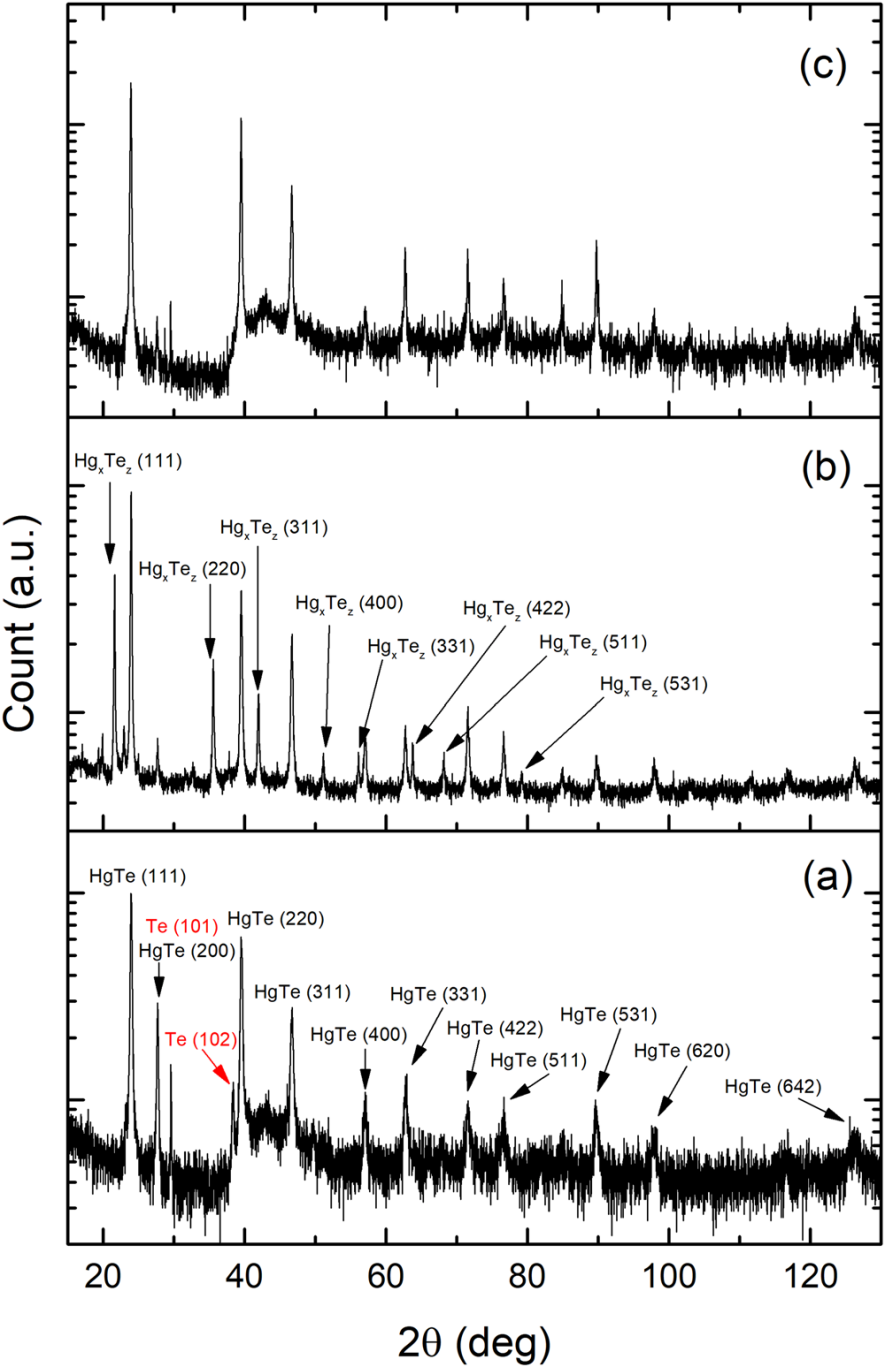
The comparison of the x-ray diffraction (XRD) results between (**a**) ingot, (**b**) SPS, and (**c**) SPS-annealing samples. The excess Te peaks in the ingot samples are highlighted by red color.

X-ray diffraction data are shown in Fig. 7. Figure 7(a) shows the original ingot contains single-phase HgTe with excess tellurium. After the SPS process, in additional to the original HgTe phase, a new crystal phase, Hg_*x*_Te_*z*_, emerges (see panel (b)), and the excess Te peaks, i.e., Te (101) and Te (102), disappear. Panel (c) shows that the SPS-annealing sample is a single-phase HgTe crystal without the excess of Te. Note that the phase of Hg_*x*_Te _*z*_ vanishes after annealing.

Additional information about the Hall coefficient measurements is provided with the supplementary material.

## Electronic supplementary material

Supplementary material
