# Supplementary material for: Semi-metals as potential thermoelectric materials
Source: Sci Rep. 2018 Jun 29;8:9876. doi: 10.1038/s41598-018-28043-3 (PMC6026171; doi:10.1038/s41598-018-28043-3)
Supplement: Supplementary file 1 — Supplementary material [file 41598_2018_28043_MOESM1_ESM.pdf]

Supplementary material for "Semi-metals as potential thermoelectric materials"

Maxime Markov[1], Xixiao Hu[2], Han-Chun Liu[1], Naiming Liu[3], S.Joseph Poon[2], Keivan Esfarjani[2,3,4], and Mona Zebarjadi *[1,3]

[1]Department of Electrical and Computer Engineering, University of Virginia, Charlottesville, Virginia 22904, USA
[2]Department of Physics, University of Virginia, Charlottesville, Virginia 22904, USA
[3]Department of Materials Science and Engineering, University of Virginia, Charlottesville, Virginia 22904, USA
[4]Department of Mechanical and Aerospace Engineering, University of Virginia, Charlottesville, Virginia 22904, USA

In this supplementary material, we provide the supporting information about the Hall coefficient measurements, the electrical conductivity fitting, the lattice thermal conductivity calculations and measurements.

S.1 Hall coefficient measurements.

In Fig. S1, we show the experimental data for the Hall effect resistance R_{xy} as a function of an applied external magnetic field B in the temperature range 200 K $\leq$ T $\leq$ 400 K. The Hall coefficient R_H can be extracted from the slope of the $R_{xy}(B)$ curve as

$$R_H(T) = \frac{R_{xy}(T)}{B} l \tag{S.1}$$

where l is the sample thickness. The net carrier concentration and electron mobilities can be found from the Hall coefficient data as

$$n(T) = \frac{1}{eR_H(T)} \tag{S.2}$$

$$\mu_e(T) = \frac{R_H(T)}{Rl} \tag{S.3}$$

and are shown in Fig. S2 and Fig. S3 respectively. Here e is an elementary charge, R is the resistance of the sample without magnetic field.

*Corresponding author. E-mail: m.zebarjadi@virginia.edu

S.2 Electrical conductivity fitting.

The electrical conductivity can be found using the following expression

$$\sigma(T,\mu) = \frac{1}{V_{cell}} \int \sigma(E) \left[-\frac{\partial f_\mu(T,E)}{\partial E} \right] dE \tag{S.4}$$

where V_{cell} is a unit cell volume, E is energy, μ is the chemical potential, f_μ is the Fermi-Dirac distribution function and $\sigma(E)$ is the differential conductivity

$$\sigma(E) = e^2 \tau(E) g(E) v_g^2(E) \tag{S.5}$$

where $g(E)$ is the density of states, $v_g(E)$ is a group velocity and $\tau(E) = 1/\Gamma(E)$ is the total relaxation time that is inversely proportional to the total scattering rate $\Gamma(E)$. In our calculations we use $g(E)$ and v_g obtained with the HSE06 exchange-correlation functional. In the constant relaxation time approximation (CRTA), one assumes that $\tau(E)$ is constant and energy independent.

In this work, we consider the energy dependent scattering rates. We consider 3 types of carrier scattering including acoustic deformation potential scattering $\Gamma^{ac}(E)$, ionized impurity scattering $\Gamma^{imp}(E)$ and polar optical scattering $\Gamma^{pop}(E)$ [3]. As follows from the Matthiessen's rule, the total scattering rate $\Gamma(E)$ is a sum of all three contributions. Overall, we have 4 fitting parameters A_1, A_2, A_3 and phonon energy $\hbar\omega$.

Acoustic deformation potential scattering rate is

$$\Gamma^{ac}(E) = A_1 g(E) \tag{S.6}$$

Ionized impurity scattering rate is

$$\Gamma^{imp}(E) = A_2 n_C T E^{-3/2} \tag{S.7}$$

where n_C is the net carrier concentration obtained from the Hall coefficient measurements (see Fig. S2)

$$n_C(T) = n_0 \exp(-T_d/T) \tag{S.8}$$

where $T_d = 450.9$ K and $n_0 = 16.01 \cdot 10^{17}$ cm^{-3}.

Polar optical scattering rate is

$$\Gamma^{pop}(E) = A_3 \frac{\hbar\omega}{v_g} \left[n_{BE}\sqrt{1+\frac{\hbar\omega}{E}} - n_{BE}\frac{\hbar\omega}{E}\sinh^{-1}\left(\sqrt{\frac{E}{\hbar\omega}}\right) + \right.$$

$$\left. +(n_{BE}+1)\sqrt{1-\frac{\hbar\omega}{E}} + (n_{BE}+1)\frac{\hbar\omega}{E}\sinh^{-1}\left(\sqrt{\frac{E}{\hbar\omega}-1}\right)\right] \tag{S.9}$$

where n_{BE} is the Bose-Einstein distribution function, v_g is the group velocity. The first two terms represent the polar-optical absorption while the last two terms describe the emission.

The energy dependent scattering rates obtained from the fitting to experimental electrical conductivity for the samples before and after annealing are shown in Fig. S4.

S.3 Thermal conductivity measurements.

To obtain the thermal conductivity κ, we use the following formula

$$\kappa(T) = \rho c_p(T) D(T) \tag{S.10}$$

where ρ is the measured density of a sample, $D(T)$ is the measured thermal diffusivity and $c_p(T)$ is the theoretical specific heat capacity. The measured thermal diffusivity for the original ingot sample and the sample after the SPS is shown in Fig. S5. The ingot sample has an excess of Te atoms, and a lower density, ρ =7.82 ± 0.04 g/cm^3, comparing to ρ = 7.98 ± 0.17 g/cm^3 after the SPS. The thermal diffusivity is higher for the ingot samples (black circles) than in the SPS samples (blue triangles), but does not change after the annealing of the SPS sample. The theoretical heat capacity is

$$c_p = c_v + V\frac{\alpha^2}{\beta_T}T \tag{S.11}$$

where α is the coefficient of thermal expansion, β_T is the isothermal diffusivity, c_V can be found from the Debye model

$$c_v = 9N_A k_B \left(\frac{T}{T_D}\right)^3 \int_0^{x_D} dx \frac{x^4 e^x}{(e^x - 1)^2} \tag{S.12}$$

where T_D = 140 K is the Debye temperature. For the second term in Eq. S.11, we use the experimental values from Ref. [2] and get the following expression for the specific heat

$$c_p(T) = c_V(T) + 1.01 \cdot 10^{-2} T \tag{S.13}$$

The obtained heat capacity $c_p(T)$ linearly changes from 0.158 JK^{-1}g^{-1} at T = 250 K to 0.171 JK^{-1}g^{-1} at T = 700 K

S.4 Isotopic scattering for phonons.

In this work, we perform *ab initio* calculations solving the Boltzmann Transport Equation (BTE). The algorithm we use is described in details in Ref. [1]. Apart from the intrinsic three-phonon scattering processes, we include the isotopic disorder scattering processes with rates given by

$$P^{iso}_{\mathbf{q}j} = \frac{\pi}{2N_{\mathbf{q}}}\omega_{\mathbf{q}j}\omega_{\mathbf{q}'j'}\delta(\hbar\omega_{\mathbf{q}j} - \hbar\omega_{\mathbf{q}'j'})\left[n_{\mathbf{q}j}n_{\mathbf{q}'j'} + \frac{n_{\mathbf{q}j} + n_{\mathbf{q}'j'}}{2}\right]\sum_s g^s_2 \left|\sum_\alpha z^{s\alpha}_{\mathbf{q}j} z^{s\alpha}_{\mathbf{q}'j'}\right|^2 \tag{S.14}$$

where $\mathbf{q}$ - phonon wave vector, j - phonon branch index, $\omega_{\mathbf{q}j}$ - frequency of phonon $(\mathbf{q}, j)$, $n_{\mathbf{q}j}$ - Bose-Einstein distribution function, α - Cartesian coordinate, s - atom type, $z^{s\alpha}_{\mathbf{q}j}$ - phonon eigenmode, g^s_2 - isotopic fluctuation parameter

$$g^s_2 = \frac{\sum_i c_i M_i^2 - \left(\sum_i c_i M_i\right)^2}{\left(\sum_i c_i M_i\right)^2} \tag{S.15}$$

We use the natural isotopic composition of Hg and Te as summarized in Table 1. The resulting isotopic fluctuation parameters are $g^s_2 = 6.5 \cdot 10^{-5}$ for Hg and $g^s_2 = 28.4 \cdot 10^{-5}$ for Te.

M_{Hg}, amu	%	M_{Te}, amu	%
195.966	0.15	119.904	0.09
197.967	9.97	121.903	2.55
198.968	16.87	122.904	0.89
199.968	23.10	123.903	4.74
200.970	13.18	124.904	7.07
201.971	29.86	125.903	18.84
203.973	6.87	127.904	31.74
		129.906	34.08

Table 1: List of natural isotopes of Hg and Te.

S.5 Accumulated thermal conductivity.

The lattice thermal conductivity can be written as

$$\kappa_L = \frac{1}{k_B T^2 V_{cell} N_q} \sum_{\nu} n_\nu (1 + n_\nu) \omega_\nu^2 c_\nu F_\nu \tag{S.16}$$

where V_{cell} is the unit cell volume, $\nu = \{\mathbf{q}, j\}$, c_ν is the group velocity, F_ν is the linear deviation of the out-of-equilibrium phonon distribution n_ν^{out} from its equilibrium value n_ν

$$n_\nu^{out} = n_\nu - \mathbf{F}_\nu \cdot \nabla T \frac{\partial n_\nu}{\partial T} \tag{S.17}$$

It can be found from the solution of the Boltzmann Transport Equation. In the relaxation time approximation (RTA) $F_\nu^{RTA} = \Lambda_\nu^{RTA} = \tau_\nu c_\nu$. In the exact solution it plays a role of a vectorial mean free-path displacement. To find a scalar mean-free path Λ_ν^{exact}, one needs to project it onto velocity direction

$$\Lambda_\nu^{exact} = \frac{\mathbf{F}_\nu \cdot \mathbf{c}_\nu}{|\mathbf{c}_\nu|} \tag{S.18}$$

The lattice thermal conductivity can be rewritten as a function of one single variable Λ as

$$\kappa_L = \sum_\nu \kappa_L(\Lambda_\nu) = \int d\Lambda \kappa_L^{acc}(\Lambda) \tag{S.19}$$

where the accumulated thermal conductivity is defined as

$$\kappa_L^{acc}(\Lambda) = \sum_\nu \kappa_L(\Lambda) \delta(\Lambda - \Lambda_\nu) \tag{S.20}$$

In Fig. S6 we show the difference in the accumulated thermal conductivities in the two approaches discussed above. As one can see, the mean free path distribution in the exact approach is shifted toward the longer values.

S.6 Summary of experimental results

In Fig. S7, we summarize the experimental data on electrical conductivity (top panel), Seebeck coefficient (middle panel) and thermal conductivity (bottom panel) in the temperature range between 300 K and 500 K. The measurements

performed on ingot and coarse powder samples (black filled circles and red empty squares correspondingly) show essentially the same thermoelectric properties. While measurements performed on annealed fine powder samples (blue filled triangles), show the similar Seebeck coefficient and thermal conductivity but not the electrical conductivity. The latter is revealed to be extremely sensitive to the number of external impurities such as excess Te atoms. One can get rid of the impurities by annealing of the samples in the presence of Hg gas. The results from the literature indicate that in this case the electrical conductivity could be enhanced almost twice. For instance, Dziuba *et al.* reported $\sigma = 1590$ S/cm [4] for the intrinsic samples (concentration is not mentioned) at T = 300 K and Whitsett *et al.* reported $\sigma = 1700$ S/cm at $p = 5.0 \cdot 10^{16}$ cm^3 and $\sigma = 1300$ S/cm at $p = 3.5 \cdot 10^{17}$ cm^3 [5]. Thus, the increase of the electrical conductivity via purification of intrinsic HgTe samples should be considered as the route to enhance the thermoelectric properties of bulk HgTe.

S.7 Seebeck coefficient convergence

In Fig. S8, we show the theoretical Seebeck coefficient of HgTe as a function of carrier concentration for $n-$ and $p-$type samples of HgTe calculated at different initial DFT k-point grids in the CRTA. We use the GGA exchange-correlation functional and the interpolated grid 20 times denser than DFT grid for this demonstration. While the full convergence is achieved at very dense grid 40x40x40 (black solid line), the coarser grid 20x20x20 has a maximum error of about $\pm 12\mu$V/K at concentrations corresponding to the maximum of the Seebeck coefficient. This coarser grid also reproduces well the positions of the Seebeck coefficient maxima as well as the overall Seebeck coefficient profile as a function of a carrier concentration. We note that our converged GGA results are consistent with the ones from the literature Ref. 15 where WIEN2K + GGA functional + BoltzTraP were used to calculate the transport properties of HgTe in the CRTA. The use of a coarser grid 20x20x20 for the calculations with HSE06 functional is enforced by its high computational cost.

References

[S1] G. Fugallo, M. Lazzeri, L. Paulatto, and F. Mauri. *Ab initio* variational approach for evaluating lattice thermal conductivity. *Phys. Rev. B*, 88:045430, 2013.

[S2] V. M. Glazov and L. M. Pavlova. *Rus. J. Phys. Chem.*, 70:441, 1996.

[S3] M. Lundstrom. *Fundamentals of carrier transport.* Cambridge University Press, New York, 2000.

[S4] Z. Dziuba and Z. T. The electrical and thermoelectrical properties of HgTe in the temperature range of intrinsic conductivity. *Physica Status Solidi*, 7:1019, 1964.

[S5] C. R. Whitsett and D. A. Nelson. Lattice thermal conductivity of p-type mercury telluride. *Physical Review B*, 5:3125, 1972.

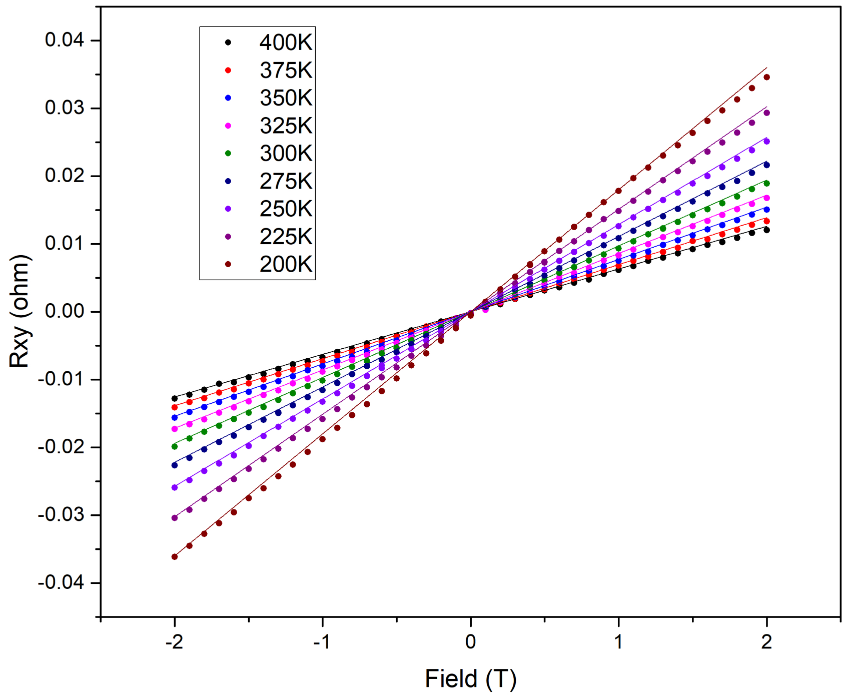

Figure S1: The Hall effect resistance R_{xy} measured as a function of magnetic field B at different temperatures.

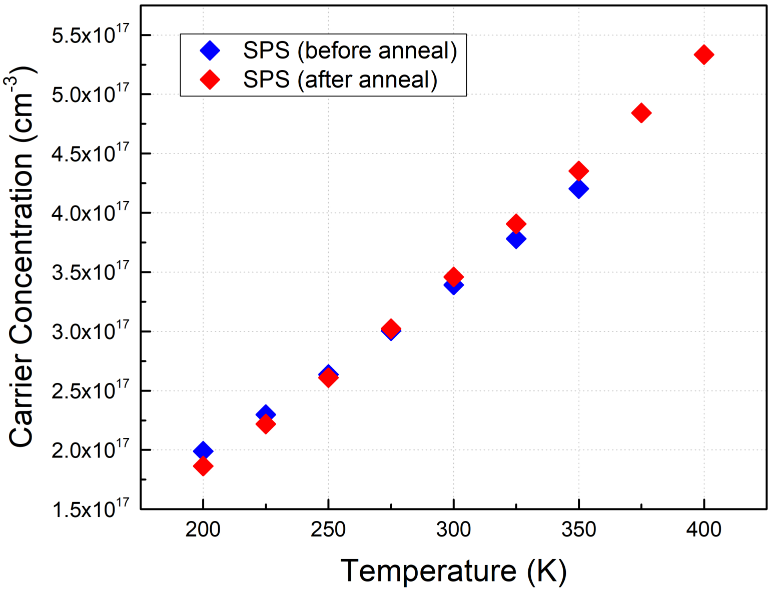

Figure S2: The net carrier concentration obtained from the Hall coefficient measurements as a function of temperature for the samples before (blue diamonds) and after (red diamonds) annealing. The samples are found to be n-type.

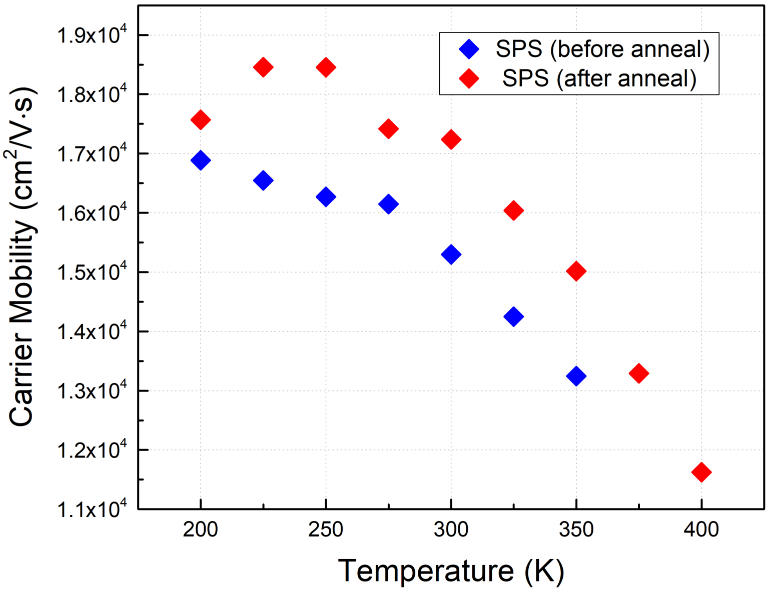

Figure S3: The experimental carrier mobilities as a function of temperature for the samples before (blue diamonds) and after (red diamonds) annealing. The mobilities are improved after annealing. In both samples, the mobilities decrease with temperature.

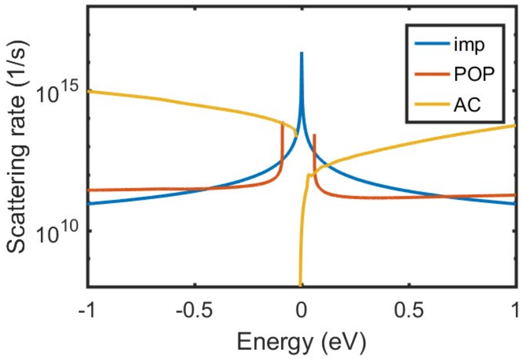

Figure S4: Acoustic deformation potential (yellow curves), polar optical (maroon curves) and charged impurity (blue curves) scattering rates obtained from the fitting of experimental electrical conductivities in the samples after annealing.

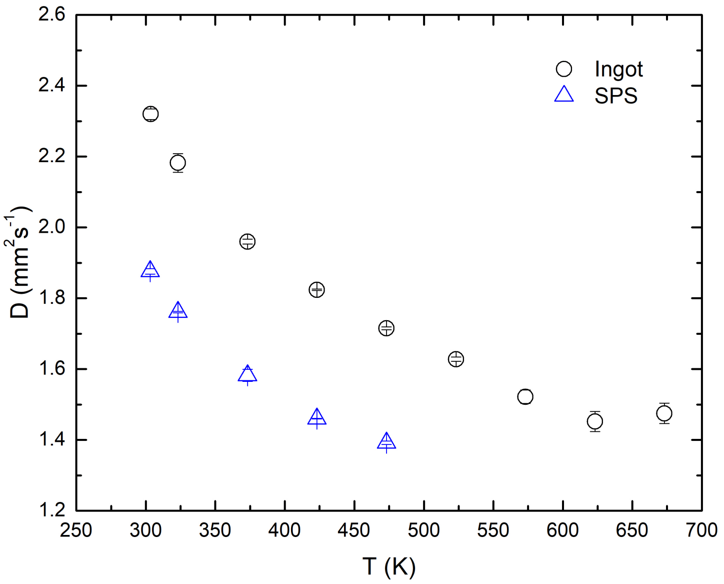

Figure S5: The temperature-dependent thermal diffusivity of HgTe ingot and SPS samples. The thermal diffusivity decreases after the SPS process, and both of ingot and SPS samples' thermal diffusivity reduce with increased temperature.

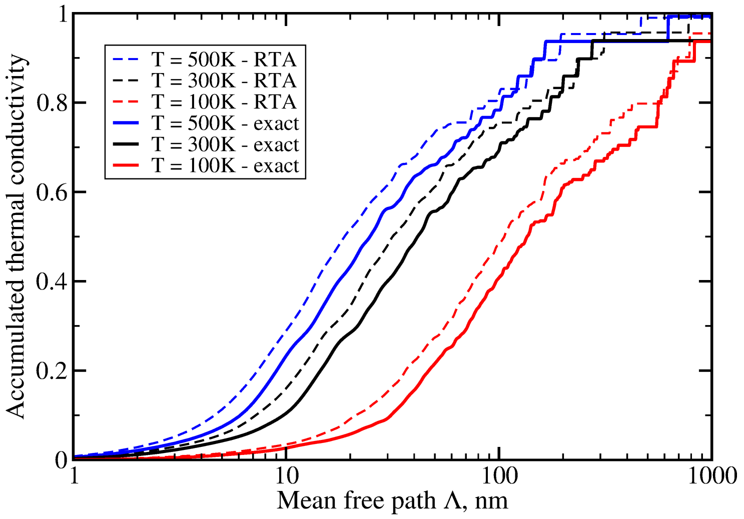

Figure S6: Accumulated thermal conductivity calculated within the RTA (dashed lines) and from the exact solution of the BTE (solid lines).

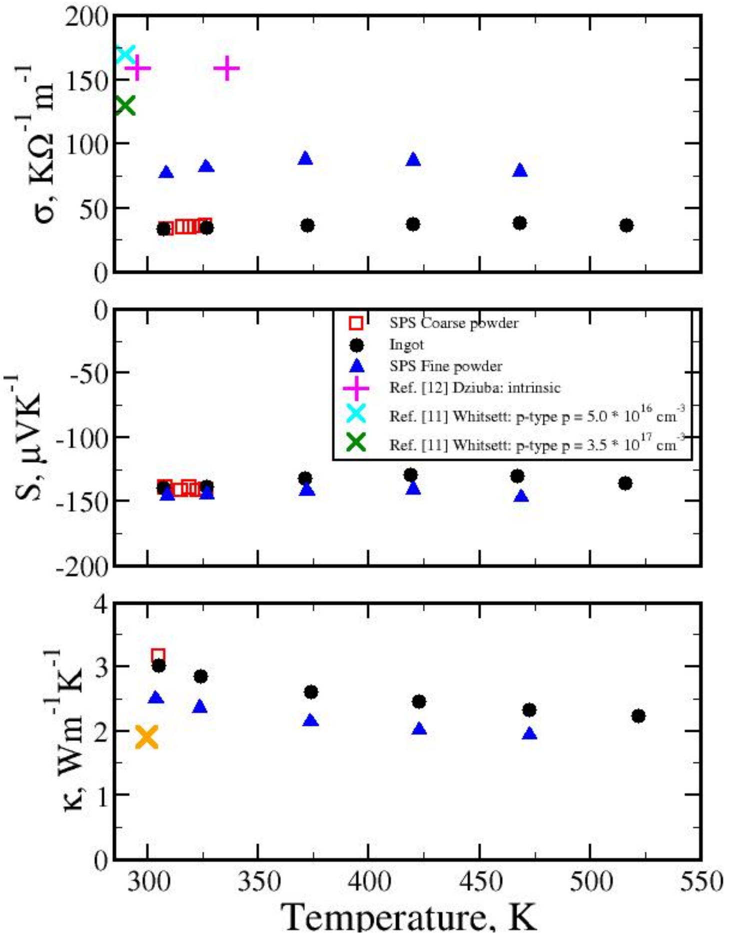

Figure S7: The summary of experimental electrical conductivity (top panel), Seebeck coefficient (middle panel) and thermal conductivity (bottom panel) as a function of temperature. Our measurements in the ingot, SPS coarse and SPS fine (annealed) samples are shown as black filled circles, red unfilled squares and blue filles triangles. The data from Ref. [11] and Ref. [12] from the main text are shown as green and cyan crosses and magenta pluses correspondingly. Yellow cross shows the data from Ref. [14] of the main text.

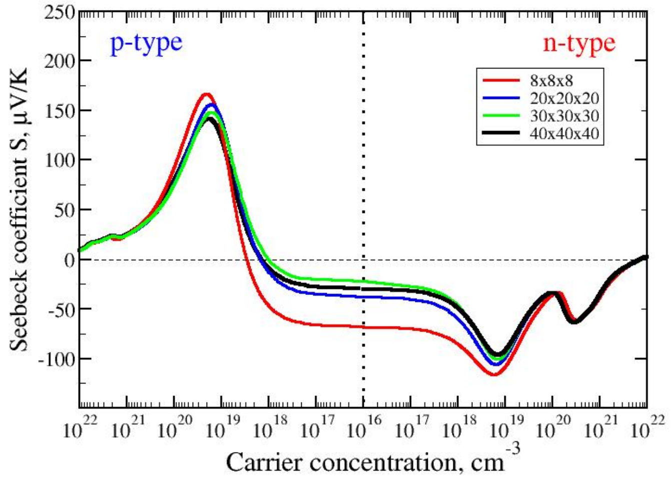

Figure S8: The Seebeck coefficient as a function of a carrier concentration for $n-$ and $p-$type samples calculated with the GGA functional in the CRTA. Different curves represent different initial DFT k-point grids: red curve - 8x8x8, blue curve - 20x20x20, green curve - 30x30x30, black curve - 40x40x40. The interpolated k-point grid is set to be 20 times more dense than the initial grid.
